# Changes in Serum and Urinary Potassium Handling Associated with Renin-Angiotensin-Aldosterone System Inhibitors in Advanced Chronic Kidney Disease Patients

**DOI:** 10.7759/cureus.5561

**Published:** 2019-09-04

**Authors:** Yuichiro Ueda, Susumu Ookawara, Haruhisa Miyazawa, Kiyonori Ito, Keiji Hirai, Taro Hoshino, Yoshiyuki Morishita

**Affiliations:** 1 Internal Medicine, First Department of Integrated Medicine, Saitama Medical Center, Saitama, JPN; 2 Nephrology, First Department of Integrated Medicine, Saitama Medical Center, Jichi Medical University, Saitama, JPN; 3 Nephrology, First Department of Integrated Medicine, Saitama Medical Center, Saitama, JPN; 4 Nephrology, Department of Internal Medicine, Saitama Red-Cross Hospital, Saitama, JPN

**Keywords:** acid-base metabolism, chronic kidney disease, diuretics, renin-angiotensin-aldosterone system inhibitors, serum potassium concentration, urinary potassium excretion

## Abstract

Objective

This study aimed to (i) compare the extent of urinary potassium (K^+^) excretion in addition to the changes in serum K^+^ concentration: and (ii) clarify the association between changes in serum K^+^ concentration, urinary K^+^ excretion, and acid-base status with or without renin-angiotensin-aldosterone system (RAAS) inhibitors in patients with advanced chronic kidney disease (CKD) stages.

Methods

Six hundred and ninety-one patients with advanced CKD (CKD G3b, 161; G4, 271; G5, 259) were retrospectively evaluated. Differences in serum K^+^ concentration, urinary K^+^ excretion, and serum sodium and chloride differences (Na^+^−Cl^-^) were compared among patients with RAAS inhibitors, RAAS inhibitors and diuretic agents, and without either medication in each CKD stage.

Results

Serum K^+^ concentrations in patients with RAAS inhibitors were significantly higher than in those with RAAS inhibitors and diuretics in CKD stage G3b and the other two treatment groups in CKD stage G4. Urinary K^+^ excretion among the three groups did not differ significantly in each CKD stage. Serum Na^+^−Cl^-^ differences in patients with RAAS inhibitors were significantly smaller than in those with RAAS inhibitors and diuretics in CKD stages G3b (*p* = 0.006) and the other two groups in CKD stage G4 (vs. RAAS inhibitors and diuretics, p <0.001; vs. without either medication, *p* = 0.008).

Conclusion

Our study demonstrated that RAAS inhibitor use might be associated with hyperkalemia via not decreased urinary K^+^ excretion but rather K^+^ redistribution from intracellular to extracellular fluid induced by the progression of metabolic acidosis in patients with advanced CKD, particularly stages G3b and G4.

## Introduction

Mineral and electrolyte disorders frequently accompany the progression of chronic kidney disease (CKD). Regarding the dysregulation of potassium (K+) homeostasis in CKD patients, careful attention should be paid in the clinical setting because of lethal arrhythmias and electrocardiographic changes [1-3]. Increase in serum K+ concentration is known to be associated with a decrease in urinary K+ excretion induced by the estimated glomerular filtration rate (eGFR) decrease, and K+ redistribution from intracellular to extracellular fluid via the progression of metabolic acidosis induced by impaired renal acid excretion [4-6].

In the treatment strategy of CKD patients, renin-angiotensin-aldosterone system (RAAS) inhibitors, including angiotensin-converting enzyme inhibitors and angiotensin receptor blockers, are usually administered to improve arterial hypertension and intraglomerular hypertension, which preserves or improves renal function [7-9]. However, the RAAS plays a key role in regulating renal K+ handling, and RAAS blockade was reportedly associated with the increase of serum K+ concentrations in CKD patients [10]. In addition, diuretic agents, including loop and thiazide diuretics, are used to adequately manage the body-fluid condition of patients with advanced CKD, and it would be possible to influence the serum K+ concentration by changing renal K+ handling induced by diuretics use [11].

However, to date, few studies have investigated the changes in urinary K+ excretion in addition to those in serum K+ concentration influenced by the use of RAAS inhibitors and /or diuretics in advanced CKD patients. Therefore, this study aimed to (i) compare the extent of urinary K+ excretion and serum K+ concentration and (ii) clarify the association between changes in serum K+ concentration, changes in urinary K+ excretion, and acid-base status with or without RAAS inhibitor and/or diuretics use in each advanced CKD stage.

## Materials and methods

This cross-sectional study included 691 patients (453 men, 238 women; mean age, 64.7 ± 0.5 years) who met the following criteria: (i) presence of CKD categorized into stages G3b-G5 according to the CKD guidelines edited by the Japanese Society of Nephrology at Saitama Medical Center, Jichi Medical University between January 2006 and December 2010; (ii) completion of urinary electrolyte measurements including K+ concentration via 24-hour urine collection; (iii) absence of dialysis treatment; and (iv) receiving RAAS inhibitors and/or diuretics including loop and thiazide diuretics [12]. The exclusion criterion was (i) receiving only diuretics because few patients received only that agent for CKD management. This study was approved by the institutional review board of Saitama Medical Center, Jichi Medical University, Japan (approval number RIN-13-30) and conforms to the provisions of the Declaration of Helsinki (as revised in Tokyo, 2004).

We collected and retrospectively analyzed sociodemographic patient data including age, sex, presence of diabetes mellitus (DM), and antihypertensive treatment with RAAS inhibitors, including angiotensin-converting enzyme inhibitors and angiotensin receptor blockers, diuretic agents, and polystyrene sulfonates for the treatment of hyperkalemia. Furthermore, we calculated the eGFR using the following equation [13]:

eGFR (mL/min/1.73 m^2^)

= 194 × age^-0.287 ^× serum creatinine^-1.094 ^(for men)

= 194 × age^-0.287 ^× serum creatinine^-1.094 ^× 0.739 (for women)

The 24-hour urine collection was performed for each patient to evaluate urinary K+ excretion. The 24-hour urine collection was initiated after the first-morning urine was discarded in the patient’s toilet. Thereafter, the entire volume of urine was collected in a disposable 3L container. To avoid the possibility of inadequate urine collection, we trained all patients to properly collect their urine samples and reinforced that 24-hour urine collection must be initiated at a specific time and then completed at the same time the next day.

Thereafter, patients included in this study were divided into the following three groups: (1) those receiving RAAS inhibitors, (2) those receiving RAAS inhibitors and diuretic agents, and (3) those not receiving RAAS inhibitors or diuretic agents. We compared clinical parameters, including serum K+ concentration and 24-hour urinary K+ excretion, in each group at each CKD stage.

Data are expressed as means ± standard error. A chi-square test was used to assess the associations between categorical variables, including the utilization and distribution of RAAS inhibitors and diuretics, complemented by an adjusted residual analysis. The differences in serum K+ concentrations, urinary K+ excretion, and serum Na+−Cl- differences among the three groups (RAAS inhibitors, RAAS inhibitors and diuretics, neither medication) in each CKD stage were evaluated by analysis of variance and Tukey’s test. All analyses were performed using SPSS Statistics for Windows version 19.0 (IBM). The level of significance was set at p < 0.05.

## Results

Patient demographics and clinical characteristics are shown in Table 1. The number of patients at each CKD stage was as follows: G3b, 161; G4, 271; G5, 259. According to CKD stage progression, the proportion of patients with DM and those using polystyrene sulfonates increased significantly in this study. Furthermore, serum K+ concentration significantly increased and urinary K+ excretion significantly decreased as CKD progressed.

**Table 1:** Patient demographics and clinical characteristics CKD, chronic kidney disease; eGFR, estimated glomerular filtration rate

CKD stage	all	G3b	G4	G5	p-value
n	691	161	271	259	
Women/Men (n)	238/453	58/103	83/188	97/162	0.227
Age (years)	64.7 ± 0.5	63.6 ± 1.1	65.0 ± 0.8	65.2 ± 0.8	0.398
With/without DM, n (%)	248/443 (35.9)	39/122 (24.2)**	101/170 (37.3)	108/151 (42.1)*	*< 0.05 vs. G4, **< 0.01 vs. G4
With/without polystyrene sulfonate, use (%)	61/630 (8.8)	6/155 (3.7)*	17/254 (6.3)	38/221 (14.7)*	*<0.01 vs. G4
eGFR (mL/min/1.73 m^2^)	21.0 ± 0.4	37.5 ± 0.3*	21.9 ± 0.3*	9.8 ± 0.2*	*< 0.001 vs. each group
Serum Na^+^ concentration (mEq/L)	139.9 ± 0.1	140.7 ± 0.2*	140.3 ± 0.2*	139.1 ± 0.2	*< 0.001 vs. G5
Serum K^+^ concentration (mEq/L)	4.69 ± 0.02	4.42 ± 0.04	4.72 ± 0.04*	4.81 ± 0.05*	*< 0.001 vs. G3b
Serum Cl^-^ concentration (mEq/L)	107.5 ± 0.2	106.3 ± 0.3	107.7 ± 0.2*	108.1 ± 0.3**	* 0.002 vs. G3b **< 0.001 vs. G3b
Urinary K^+^ excretion (mEq/day)	32.1 ± 0.6	40.5 ± 1.3*	34.0 ± 0.9*	24.7 ± 0.8*	*< 0.001 vs. each group

In addition, there were no differences in eGFR values among patients treated with RAAS inhibitors, with RAAS inhibitors and diuretics, or without either medication at each CKD stage, except for patients with CKD stage G4 treated with RAAS inhibitors and diuretics, and those treated without either medication (Table 2). The proportions of patients with DM were significantly larger in the group treated with RAAS inhibitors and diuretics than in the other two groups.

**Figure 1:**
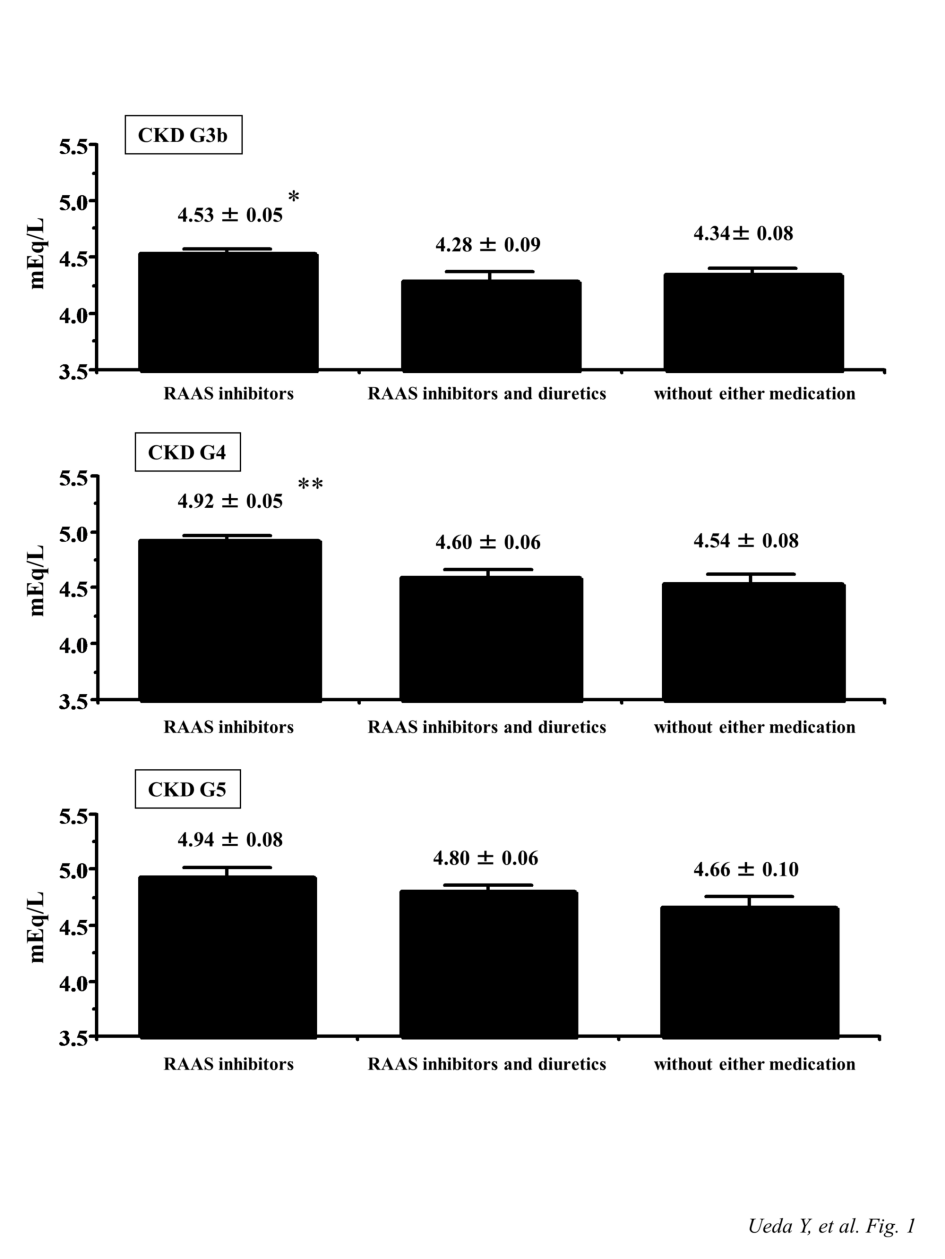
Comparison of serum K+ concentrations among patients with CKD stage G3b, G4, and G5 treated with RAAS inhibitors, RAAS inhibitors and diuretics, or without either medication *p < 0.05 vs. treated with RAAS inhibitors and diuretics. **p < 0.001 vs. treated with RAAS inhibitors and diuretics, and without either medication, respectively. CKD, chronic kidney disease; RAAS, renin-angiotensin-aldosterone system

As shown in Fig. 1, serum K+ concentration at CKD stage G3b was significantly higher in patients treated with RAAS inhibitors than in those treated with RAAS inhibitors and diuretics (4.53 ± 0.05 vs. 4.28 ± 0.09 mEq/L, *p* = 0.017). In addition, in patients with CKD stage G4, serum K+ concentration was significantly higher in those treated with RAAS inhibitors than those treated with RAAS inhibitors and diuretics, and those treated without either medication (RAAS inhibitors, 4.92 ± 0.05 mEq/L; RAAS inhibitors and diuretics, 4.60 ± 0.06 mEq/L; without either medication; 4.54 ± 0.08 mEq/L; RAAS inhibitors vs. other groups, *p* < 0.001). However, in patients with CKD stage G5, there were no significant differences in serum K+ concentration among the three groups.

**Figure 2:**
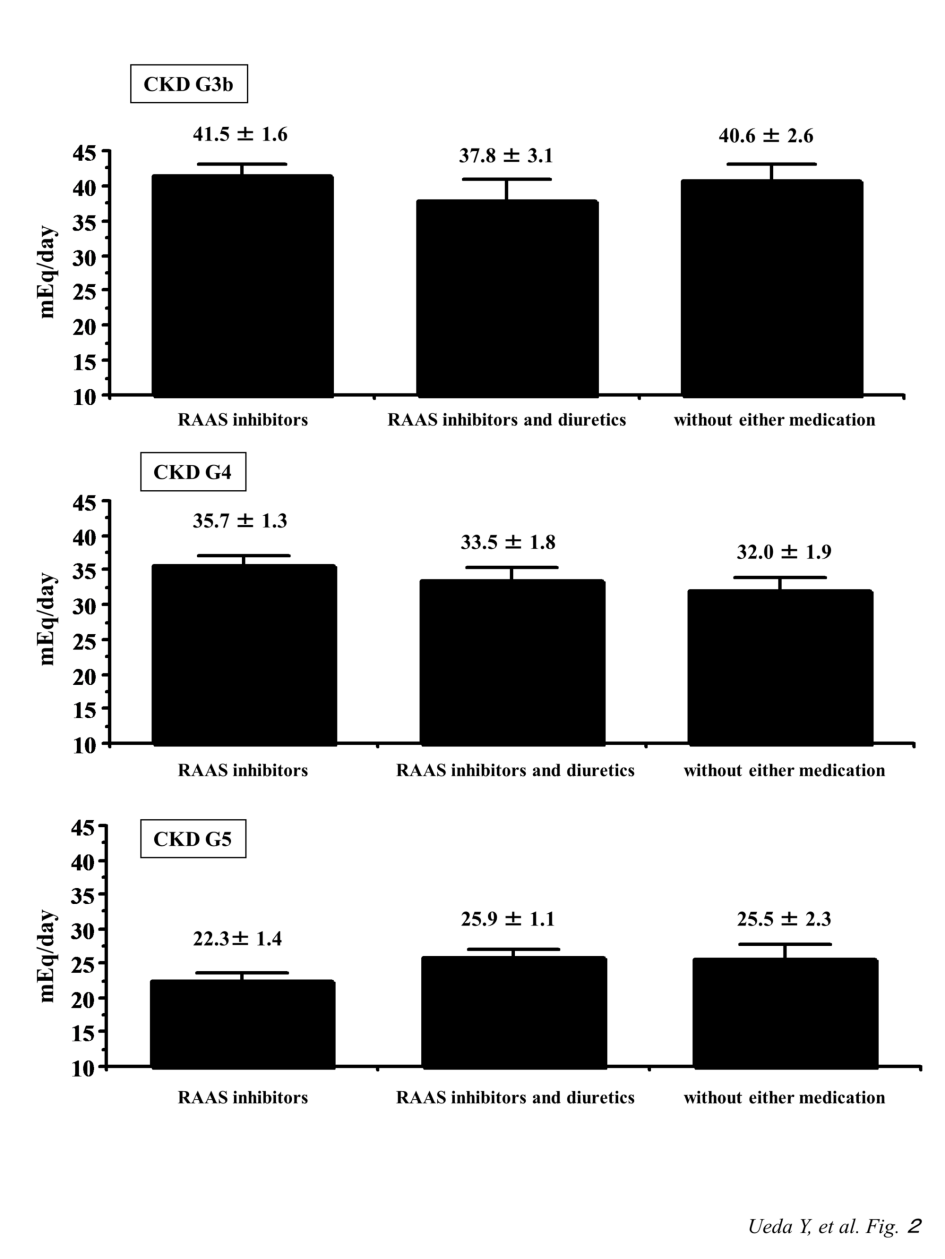
Comparison of urinary K+ excretion among patients with CKD stage G3b, G4, and G5 treated with RAAS inhibitors, with RAAS inhibitors and diuretics, or without either medication CKD, chronic kidney disease, RAAS, renin-angiotensin-aldosterone system

Figure 2 shows the changes in urinary K+ excretion among the three groups by each CKD stage. In the advanced CKD stages including stage G3b, G4, and G5 in this study, there were no differences in urinary K+ excretions among the three groups.

However, differences of Na+ and Cl- concentration in blood of patients treated RAAS inhibitors were significantly smaller than those of patients with CKD stage G3b and G5 treated with RAAS inhibitors and diuretics (CKD stage G3b, *p* = 0.006; CKD stage G5, *p* = 0.008) and the other two groups, respectively, of patients with CKD G4 (vs. RAAS inhibitors and diuretics, *p *< 0.001; vs. without either medication, *p* = 0.008; Table 3).

**Table 2:** Comparisons of serum Na+ and Cl- concentrations and differences among patients with CKD stage G3b, G4, and G5 treated with RAAS inhibitors, with RAAS inhibitors and diuretics, or without either medication CKD, chronic kidney disease; RAAS, renin-angiotensin-aldosterone system

		RAAS inhibitors	RAAS inhibitors and diuretics	without either medication	p -value
CKD G3b	n	81	35	45	
	Serum Na^+^ concentration (mEq/L)	140.5 ± 0.3	141.0 ± 0.4	140.8 ± 0.4	0.488
	Serum Cl^-^ concentration (mEq/L)	106.4 ± 0.4	105.2 ± 0.5	107.0 ± 0.5	0.067
	Serum Na^+^−Cl^-^ differences (mEq/L)	34.0 ± 0.3*	35.9 ± 0.5	33.9 ± 0.4**	* 0.006 vs. RAAS inhibitors and diuretics ** 0.008 vs. RAAS inhibitors and diuretics
CKD G4	n	120	94	57	
	Serum Na^+^ concentration (mEq/L)	140.0 ± 0.3	140.3 ± 0.3	141.0 ± 0.4	0.075
	Serum Cl^-^ concentration (mEq/L)	108.4 ± 0.3*	106.7 ± 0.4	107.9 ± 0.4	* 0.002 vs. RAAS inhibitors and diuretics
	Serum Na^+^ - Cl^-^ differences (mEq/L)	31.6 ± 0.3*^,^**	33.6 ± 0.3	33.1 ± 0.5	*< 0.001 vs. RAAS inhibitors and diuretics ** 0.008 vs. without either medication
CKD G5	n	72	147	40	
	Serum Na^+^ concentration (mEq/L)	139.9 ± 0.3	138.9 ± 0.4	138.4 ± 0.7	0.089
	Serum Cl^-^ concentration (mEq/L)	109.9 ± 0.5*	107.2 ± 0.4	108.3 ± 0.8	* 0.001 vs. RAAS inhibitors and diuretics
	Serum Na^+^ - Cl^-^ differences (mEq/L)	30.0 ± 0.4*	31.7 ± 0.3	30.1 ± 0.6	* 0.008 vs. RAAS inhibitors and diuretics

## Discussion

The evaluation of K+ metabolism disorders is one of the most important considerations in patients with advanced CKD. In particular, hyperkalemia is the most arrhythmogenic and lethal form of an electrolyte disorder and has multifactorial causes [1-3]. Severe hyperkalemia, defined as serum K+ concentration more than 6.5 mEq/L, affected 0.64% of patients who underwent hospital admission, and 70% of these patients had comorbid CKD [14]. Furthermore, according to the previous retrospective studies, the increase in serum K+ concentration was independently associated with the deterioration of renal function, DM, elderly age, and RAAS inhibitor use [15-16]. In this study, serum K+ concentration of patients treated with RAAS inhibitors was significantly higher than that of those treated with RAAS inhibitors and diuretics or treated without either medication in CKD stages G3b and G4, although the proportion of DM presence in the RAAS inhibitors treatment group was not larger than those of the other two groups. However, in patients with CKD stage G5, there were no significant differences in serum K+ concentration among the three groups. Based on this result, in patients with severely advanced CKD such as CKD stage G5, the use of RAAS inhibitors and/or diuretics might not influence the regulation of serum K+ concentration anymore, and the deterioration of renal function itself would be mainly associated with serum K+ increases beyond the use of these agents.

In the renal regulation of K+ homeostasis, the effect of aldosterone is the main factor influencing urinary K+ excretion [17]. Aldosterone stimulates Na+ reabsorption at the distal nephron, including principal cells in the cortical collecting duct (CCD), by increasing Na+ transport through epithelial Na+ channels. The excretion of K+ in the principal cells is mainly mediated by apical membrane K+ channels corresponding to Na+ reabsorption via aldosterone action. Therefore, the use of RAAS inhibitors would be expected to reduce the urinary K+ excretion. However, to date, reports examining the relationship between RAAS inhibitor use and urinary K+ excretion in advanced CKD patients are limited. The present study revealed no significant differences in urinary K+ excretion with the use of RAAS inhibitors, RAAS inhibitors and diuretics, or neither medication in patients with CKD stage G3b, G4, or G5. According to the previous studies, there were no significant changes in urinary K+ excretion, transtubular K+ gradient, and fractional excretion of K+ after RAAS inhibitor administration [18-19]. Furthermore, there were no changes in urinary K+ excretion before versus after RAAS inhibitor administration (before, 2652 ± 897 mg/day; after, 2691 ± 936 mg/day) under a normal K+ intake [20]. Our results might be similar to those of that report, although the patients included in this study all had advanced CKD, and the daily K+ intake would be restricted in the clinical setting [20]. In addition, loop diuretics and thiazides are known to increase urinary K+ excretion, which would be induced by the stimulation of flow-dependent K+ secretion from the CCD [21]. However, in this study, the use of diuretics with RAAS inhibitors did not show increased urinary K+ excretion compared to that treated with RAAS inhibitors, and without either medication, respectively, in each advanced CKD stage. The influence of diuretics to the urinary K+ excretion is opposed by RAAS inhibitors; therefore, urinary K+ excretion might not be different theoretically between patients with RAAS inhibitors and diuretics, and those without either medication. However, we could hardly explain the reasons why there was no significant difference in urinary K+ excretion between patients with RAAS inhibitors and those with RAAS inhibitors and diuretics; therefore, further studies are needed regarding the association between RAAS inhibitor and/or diuretics use, and urinary K+ excretion in patients with advanced CKD stages.

The serum Na+−Cl- difference was calculated instead of serum HCO3- because of the few measurements of serum HCO3- made in this study. The relationship between serum Na+−Cl- difference, serum HCO3- level, and the anion gap can be expressed by the following equation: serum Na+−Cl- difference = serum HCO3- level + anion gap. Under the assumption that the anion gap is unchanged or changed in the same way among the three groups in each CKD stage, changes in serum Na+−Cl- difference are expected to reflect changes in serum HCO3- level. Indeed, there were reportedly significant correlations between serum HCO3- level and serum Na+−Cl- difference in patients with liver cirrhosis and in those with CKD [22-23]. Thus, decreases in serum Na+−Cl- differences would reflect decreases in serum HCO3- levels associated with metabolic acidosis. In this study, serum Na+−Cl- differences in patients treated with RAAS inhibitors were significantly smaller than those treated with RAAS inhibitors and diuretics, or those treated without either medication in each CKD stage. Acid excretion along the CCD is reportedly regulated by RAAS, and aldosterone is an important stimulator of H+-adenosine triphosphatase activity at CCD [5-6]. Furthermore, RAAS blockade impairs renal acid excretion, which leads to the progression of metabolic acidosis [24]. Therefore, our results might imply the progression of metabolic acidosis under RAAS inhibitor use, and significant increases in serum K+ concentration in patients with RAAS inhibitors in CKD stages G3b and G4 might be associated with not the decrease of urinary K+ excretion, but rather, K+ redistribution from intracellular to extracellular fluid caused by the progression of metabolic acidosis. The results in the present study were similar to those of a previous report of RAAS blockade increasing serum K+ concentration by interfering with extra-renal/transcellular K+ disposition mechanisms [25]. However, in this study, significant increases of serum Na+−Cl- differences in patients with RAAS inhibitors and diuretics were confirmed compared to those without either medication in CKD stage G3b and those with RAAS inhibitors in CKD stage G5, and these increases were considered no association with changes in serum K+ concentration in each advanced CKD stage. Therefore, further studies, including measurements of serum HCO3- concentrations, are required to clarify the relationship between changes in serum K+ concentration and acid-base status in patients with advanced CKD stages.

This study has several limitations. First, we could not evaluate the K+ intake of each patient; therefore, we cannot comment on the influence of K+ intake on changes in serum K+ concentration and urinary K+ excretion in this study. Second, serum HCO3- concentrations could not be routinely measured. Therefore, we cannot but predict each patient’s acid-base metabolism using changes in serum Na+−Cl- difference instead of changes of serum HCO3- level. Finally, we could not measure serum aldosterone concentrations; therefore, we cannot examine the relationship between serum aldosterone concentrations and renal and/or extra-renal K+ handling in this study.

## Conclusions

Our study demonstrated that RAAS inhibitor use was associated with hyperkalemia via not decreased urinary K+ excretion but rather K+ redistribution from intracellular to extracellular fluid induced by the progression of metabolic acidosis in patients with advanced CKD, particularly stages G3b and G4.
